# Supplementary material for: A UMLS-based spell checker for natural language processing in vaccine safety
Source: BMC Med Inform Decis Mak. 2007 Feb 12;7:3. doi: 10.1186/1472-6947-7-3 (PMC1805499; doi:10.1186/1472-6947-7-3)
Supplement: Additional file 13 — RAP application directory. Sets up directory for RAP (RDF application for PHP) source code [file 1472-6947-7-3-S13.gz › rap/doc/phpdoc/__filesource/fsource_ontModel__ontModelOntProperty.php.html]

xml version="1.0" encoding="iso-8859-1"?


File Source for OntProperty.php


# Source for file OntProperty.php

Documentation is available at OntProperty.php

```
1. <?PHP
2. /**
3. * ----------------------------------------------------------------------------------
4. * Class: OntProperty
5. * ----------------------------------------------------------------------------------
6. *
7. * @package     ontModel
8. */


11. /**
12. * Class encapsulating a property in an ontology.
13. *
14. * <BR><BR>History:
15. * <LI>10-05-2004                : First version of this class.</LI>
16. *
17. * @version  V0.9.1
18. * @author Daniel Westphal <mail at d-westphal dot de>
19. *
20. *
21. * @package     ontModel
22. * @access    public
23. ***/

25. class OntProperty extends OntResource
26. {
27. /**
28. * Constructor.
29. * You can supply a URI.
30. *
31. * @param string $uri
32. * @access    public
33. */
34. function OntProperty($uri = null)
35. {
36. parent::OntResource($uri);
37. }

39. /**
40. * Add a resource representing the domain of this property.
41. *
42. * @param    object ResResource    $resResource
43. * @return    boolean
44. * @access    public
45. */
46. function addDomain($resResource)
47. {
48. return $this->addProperty($this->vocabulary->DOMAIN(),$resResource);
49. }

51. /**
52. * Add a resource representing the range of this property.
53. *
54. * @param    object ResResource    $resResource
55. * @return    boolean
56. * @access    public
57. */
58. function addRange($resResource)
59. {
60. return $this->addProperty($this->vocabulary->RANGE(),$resResource);
61. }

63. /**
64. * Add a sub-property of this property.
65. *
66. * @param    object ResProperty    $resProperty
67. * @return    boolean
68. * @access    public
69. */
70. function addSubProperty($resProperty)
71. {
72. return $resProperty->addProperty($this->vocabulary->SUB_PROPERTY_OF(),$this);
73. }

75. /**
76. * Add a super-property of this property.
77. *
78. * @param    object ResProperty    $resProperty
79. * @return    boolean
80. * @access    public
81. */
82. function addSuperProperty($resProperty)
83. {
84. return $this->addProperty($this->vocabulary->SUB_PROPERTY_OF(),$resProperty);
85. }

87. /**
88. * Answer a OntClass that represents the domain class of this property.
89. * If there is more than one such resource, an arbitrary selection is made.
90. *
91. * @return    object OntClass
92. * @access    public
93. */
94. function getDomain()
95. {
96. return $this->getPropertyValue($this->vocabulary->DOMAIN(),'OntClass');
97. }

99. /**
100. * Answer a OntClass that represents the range class of this property.
101. * If there is more than one such resource, an arbitrary selection is made.
102. *
103. * @return    object OntClass
104. * @access    public
105. */
106. function getRange()
107. {
108. return $this->getPropertyValue($this->vocabulary->RANGE(),'OntClass');
109. }

111. /**
112. * Answer a property that is the sub-property of this property.
113. * If there is more than one such property, an arbitrary selection is made.
114. *
115. * @return    object OntProperty
116. * @access    public
117. */
118. function getSubProperty()
119. {
120. $statement = $this->model->findFirstMatchingStatement(null,$this->vocabulary->SUB_PROPERTY_OF(),$this);
121. if ($statement !== null)
122. return new OntProperty($statement->getLabelSubject());

124. return null;
125. }

127. /**
128. * Answer a property that is the super-property of this property.
129. * If there is more than one such property, an arbitrary selection is made.
130. *
131. * @return    object OntProperty
132. * @access    public
133. */
134. function getSuperProperty()
135. {
136. return $this->getPropertyValue($this->vocabulary->SUB_PROPERTY_OF(),'OntProperty');
137. }

139. /**
140. * Answer true if the given resource a class specifying the domain of this property.
141. *
142. * @param    object ResResource    $resResource
143. * @return    boolean
144. * @access    public
145. */
146. function hasDomain($resResource)
147. {
148. return $this->hasProperty($this->vocabulary->DOMAIN(),$resResource);
149. }

151. /**
152. * Answer true if the given resource a class specifying the range of this property.
153. *
154. * @param    object ResResource    $resResource
155. * @return    boolean
156. * @access    public
157. */
158. function hasRange($resResource)
159. {
160. return $this->hasProperty($this->vocabulary->RANGE(),$resResource);
161. }

163. /**
164. * Answer true if the given property is a sub-property of this property.
165. * If $direct is set to true, only consider the direcly adjacent
166. * properties in the property hierarchy
167. *
168. * @param    object ResResource    $resProperty
169. * @param    boolean    $direct
170. * @return    boolean
171. * @access    public
172. */
173. function hasSubProperty($resProperty, $direct = true)
174. {
175. if ($direct)
176. return $resProperty->hasProperty($this->vocabulary->SUB_PROPERTY_OF(),$this);

178. $index=array();
179. return ($this->_getSubAttributeStatementsRec($this,$this->vocabulary->SUB_PROPERTY_OF(),$index,$resProperty) === true);
180. }

182. /**
183. * Answer true if the given property is a super-property of this property.
184. * If $direct is set to true, only consider the direcly adjacent
185. * properties in the property hierarchy
186. *
187. * @param    object ResResource    $resProperty
188. * @param    boolean    $direct
189. * @return    boolean
190. * @access    public
191. */
192. function hasSuperProperty($resProperty, $direct = true)
193. {
194. if ($direct)
195. return $this->hasProperty($this->vocabulary->SUB_PROPERTY_OF(),$resProperty);

197. $index=array();
198. return ($this->_getSuperAttributeStatementsRec($this,$this->vocabulary->SUB_PROPERTY_OF(),$index,$resProperty) === true);
199. }

201. /**
202. * Answer an array of all of the declared domain classes of this property.
203. * Each element of the iterator will be an OntClass.
204. *
205. * @return    array of OntClasses
206. * @access    public
207. */
208. function listDomain()
209. {
210. return $this->listProperty($this->vocabulary->DOMAIN(),'OntClass');
211. }

213. /**
214. * Answer an array of all of the declared range classes of this property.
215. * Each element of the iterator will be an OntClass.
216. *
217. * @return    array of OntClasses
218. * @access    public
219. */
220. function listRange()
221. {
222. return $this->listProperty($this->vocabulary->RANGE(),'OntClass');
223. }

225. /**
226. * Answer an array of all the properties that are declared to be
227. * sub-properties of this property. Each element of the iterator will be an
228. * OntProperty.
229. * If $direct is set to true, only consider the direcly adjacent
230. * properties in the property hierarchy
231. *
232. * @param    boolean    $direct
233. * @return    array of OntProperties
234. * @access    public
235. */
236. function listSubProperties($direct = true)
237. {
238. $return = array();
239. if ($direct)
240. {
241. $statements = $this->model->find(null,$this->vocabulary->SUB_PROPERTY_OF(),$this);
242. } else
243. {
244. $index = array();
245. $statements = $this->_getSubAttributeStatementsRec($this,$this->vocabulary->SUB_PROPERTY_OF(),$index);
246. }

248. $returnIndex=array();
249. foreach ($statements as $statement)
250. {
251. $objectLabel=$statement->getLabelSubject();
252. if (!in_array($objectLabel,$returnIndex))
253. {
254. $returnIndex[]=$objectLabel;
255. $return[]=new OntProperty($statement->getLabelSubject());
256. }
257. }
258. return $return;
259. }

261. /**
262. * Answer an array of all the properties that are declared to be
263. * super-properties of this property. Each element of the iterator will be an
264. * OntProperty.
265. * If $direct is set to true, only consider the direcly adjacent
266. * properties in the property hierarchy
267. *
268. * @param    boolean    $direct
269. * @return    array of OntProperties
270. * @access    public
271. */
272. function listSuperProperties($direct = true)
273. {
274. $return = array();
275. if ($direct)
276. return $this->listProperty($this->vocabulary->SUB_PROPERTY_OF(),'OntProperty');

278. $index=array();
279. $statements = $this->_getSuperAttributeStatementsRec($this,$this->vocabulary->SUB_PROPERTY_OF(),$index);
280. $returnIndex=array();
281. foreach ($statements as $statement)
282. {
283. $objectLabel=$statement->getLabelObject();
284. if (!in_array($objectLabel,$returnIndex))
285. {
286. $returnIndex[]=$objectLabel;
287. $return[]=new OntProperty($statement->getLabelObject());
288. }
289. }
290. return $return;
291. }

293. /**
294. * Remove the given class from the stated domain(s) of this property.
295. *
296. * @param    object ResResource $resResource
297. * @return    boolean
298. * @access    public
299. */
300. function removeDomain($resResource)
301. {
302. return $this->removeProperty($this->vocabulary->DOMAIN(),$resResource);
303. }

305. /**
306. * Remove the given class from the stated range(es) of this property.
307. *
308. * @param    object ResResource $resResource
309. * @return    boolean
310. * @access    public
311. */
312. function removeRange($resResource)
313. {
314. return $this->removeProperty($this->vocabulary->RANGE(),$resResource);
315. }

317. /**
318. * Remove the given property from the sub-properties of this property.
319. *
320. * @param    object ResProperty $resProperty
321. * @return    boolean
322. * @access    public
323. */
324. function removeSubProperty( )
325. {
326. return    $this->model->remove(new Statement($resProperty,$this->vocabulary->SUB_PROPERTY_OF(),$this));
327. }

329. /**
330. * Remove the given property from the super-properties of this property.
331. *
332. * @param    object ResProperty $resProperty
333. * @return    boolean
334. * @access    public
335. */
336. function removeSuperProperty($resProperty)
337. {
338. return $this->removeProperty($this->vocabulary->SUB_PROPERTY_OF(),$resProperty);
339. }

341. /**
342. * Assert that the given resource represents the class of individuals
343. * that form the domain of this property. Any existing domain statements
344. * for this property are removed.
345. *
346. * @param    object ResResource $resResource
347. * @access    public
348. */
349. function setDomain($resResource)
350. {
351. $this->setPropertyValue($this->vocabulary->DOMAIN(),$resResource);
352. }

354. /**
355. * Assert that the given resource represents the class of individuals
356. * that form the range of this property. Any existing range statements
357. * for this property are removed.
358. *
359. * @param    object ResResource $resResource
360. * @access    public
361. */
362. function setRange($resResource)
363. {
364. $this->setPropertyValue($this->vocabulary->RANGE(),$resResource);
365. }

367. /**
368. * Assert that this property is super-property of the given property.
369. * Any existing statements for superPropertyOf on prop will be removed.
370. *
371. * @param    object ResProperty $resProperty
372. * @access    public
373. */
374. function setSubProperty($resProperty)
375. {
376. foreach ($this->listSubProperties() as $oldResProperty)
377. {
378. $this->removeSubProperty($oldResProperty);
379. }

381. $this->addSubProperty($resProperty);
382. }

384. /**
385. * Assert that this property is sub-property of the given property.
386. * Any existing statements for subPropertyOf on prop will be removed.
387. *
388. * @param    object ResProperty $resProperty
389. * @access    public
390. */
391. function setSuperProperty($resProperty)
392. {
393. $this->setPropertyValue($this->vocabulary->SUB_PROPERTY_OF(),$resProperty);
394. }
395. }
396. ?>
```

Documentation generated on Thu, 7 Jul 2005 13:42:25 +0200 by phpDocumentor 1.3.0RC3
